# Gonadotropin-Releasing Hormone Receptor (GnRHR) and Hypogonadotropic Hypogonadism

**DOI:** 10.3390/ijms242115965

**Published:** 2023-11-04

**Authors:** Pavlos Fanis, Vassos Neocleous, Irene Papapetrou, Leonidas A. Phylactou, Nicos Skordis

**Affiliations:** 1 Department of Molecular Genetics, Function and Therapy, The Cyprus Institute of Neurology and Genetics, Nicosia 2371, Cyprus; pavlosf@cing.ac.cy (P.F.); vassosn@cing.ac.cy (V.N.); 2 School of Medicine, University of Nicosia, Nicosia 1678, Cyprus; irenepapapetrou@hotmail.com; 3 Division of Paediatric Endocrinology, Paedi Center for Specialized Paediatrics, Nicosia 2024, Cyprus

**Keywords:** HPG axis, GnRH, GnRHR, hypogonadotropic hypogonadism, pituitary, gonadotropes

## Abstract

Human sexual and reproductive development is regulated by the hypothalamic-pituitary-gonadal (HPG) axis, which is primarily controlled by the gonadotropin-releasing hormone (GnRH) acting on its receptor (GnRHR). Dysregulation of the axis leads to conditions such as congenital hypogonadotropic hypogonadism (CHH) and delayed puberty. The pathophysiology of GnRHR makes it a potential target for treatments in several reproductive diseases and in congenital adrenal hyperplasia. GnRHR belongs to the G protein-coupled receptor family and its GnRH ligand, when bound, activates several complex and tissue-specific signaling pathways. In the pituitary gonadotrope cells, it triggers the G protein subunit dissociation and initiates a cascade of events that lead to the production and secretion of the luteinizing hormone (LH) and follicle-stimulating hormone (FSH) accompanied with the phospholipase C, inositol phosphate production, and protein kinase C activation. Pharmacologically, GnRHR can be modulated by synthetic analogues. Such analogues include the agonists, antagonists, and the pharmacoperones. The agonists stimulate the gonadotropin release and lead to receptor desensitization with prolonged use while the antagonists directly block the GnRHR and rapidly reduce the sex hormone production. Pharmacoperones include the most recent GnRHR therapeutic approaches that directly correct the misfolded GnRHRs, which are caused by genetic mutations and hold serious promise for CHH treatment. Understanding of the GnRHR’s genomic and protein structure is crucial for the most appropriate assessing of the mutation impact. Such mutations in the GNRHR are linked to normosmic hypogonadotropic hypogonadism and lead to various clinical symptoms, including delayed puberty, infertility, and impaired sexual development. These mutations vary regarding their mode of inheritance and can be found in the homozygous, compound heterozygous, or in the digenic state. GnRHR expression extends beyond the pituitary gland, and is found in reproductive tissues such as ovaries, uterus, and prostate and non-reproductive tissues such as heart, muscles, liver and melanoma cells. This comprehensive review explores GnRHR’s multifaceted role in human reproduction and its clinical implications for reproductive disorders.

## 1. Introduction

Reproduction and development of sexual characteristics, in humans, is under the control of the hypothalamic-pituitary-gonadal (HPG) axis. Gonadotropin-releasing hormone (GnRH) that is produced in the hypothalamic neurosecretory cells is the main regulatory hormone of the HPG axis and acts in a pulsatile manner on the gonadotrope cells of the anterior pituitary gland by binding to GnRH receptors (GnRHRs) [1,2,3]. The stimulated GnRHRs cause the production and secretion of the luteinizing hormone (LH) and the follicle-stimulating hormone (FSH) that are known to control several important functions and have a direct effect on the gonads. Such functions include the gonadal steroid production, gametogenesis, and the gonadal cell proliferation [4,5,6]. Stimulation of the GnRHR by the GnRH takes place through the G_q/11_ heterotrimeric protein pathway [4,5,7]. During childhood, GnRH secretion is suppressed and rises during puberty, in which increased production of gonadotropins and gonadal steroids trigger sexual development [8]. Reproduction development is disrupted somewhat by dysfunction of the GnRHR including causative mutations in the *GnRHR* gene resulting in the delay of puberty and congenital hypogonadotropic hypogonadism (CHH) [9,10]. In humans, GnRHRs are divided into two subtypes, GnRH1R and GnRH2R, with the expression of the latter being controversial [11,12,13]. The GnRH1R receptor is a member of the rhodopsin G protein-coupled receptor (GPCR) family and, in addition to its primary expression in pituitary gonadotrope cells, it was also found to be expressed in the breast, ovaries, prostate cells and lymphocytes [14,15,16,17]. Given its importance in reproductive regulation, the GnRHR has emerged as a potential target for the treatment of infertility and sex steroid-dependent hyperplasia, such as uterine fibroids, endometriosis, and prostate cancer [18,19,20,21]. In these conditions, gonadal steroid secretion is reduced by the delivery of GnRH antagonists or high doses of GnRH agonists, which reduce the expression of the receptor [22,23]. In this review, the main type of human GnRHR receptor is outlined and its main role in regulating reproduction through complex signaling pathways is described. Additionally, the genomic and protein structure of GNRHR along with ligands, mutations, and therapeutic possibilities are also explored. A discussion on tissue expression patterns of GNRHR, extending beyond the pituitary gland, and their possible implication on clinical entities such as hypogonadotropic hypogonadism is also presented.

## 2. Genomic and Protein Structure of GnRHR

### 2.1. Genomic Structure of GnRHR

**Figure 1:**
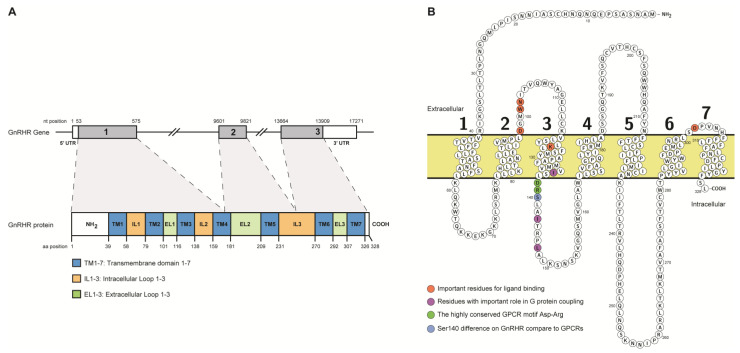
Gene and protein structure of GnRHR. (**A**) Schematic representation of the GNRHR gene and protein. Exon/Intron organization on the gene and the amino-terminal (NH_2_) tail, transmembrane (TM), intracellular (IL), extracellular (EL), and carboxy-terminal (COOH) domains on protein are indicated. The three exons of gene are indicated with numbers 1–3. (**B**) The GnRHR structural organization in the pituitary gonadotrope cell membrane. Ligand-binding amino acid residues are indicated with orange color, G protein coupling amino acid residues are indicate with purple color, the highly conserved Asp-Arg residues are indicated with green color, and the Ser140 which is different compare to other GPCRs, is indicated with blue color. The seven transmembrane domains of the protein are indicated with numbers 1–7.

Using cloning and mapping analyses, the human GnRH receptor gene was found to be located on chromosome 4q13.2–13.3 and consists of three exons separated by two introns covering 17.2 kb on the chromosome [24,25,26,27]. Specifically, the chromosomal location is Chromosome 4: 67,737,118–67,754,388 (genome assembly: GRCh38.p14); gene code (Gene: ENSG00000109163.7). The main transcript of the gene is 4402 bp in length and encodes a protein of size 328 aa and predicted unmodified molecular weight of ~38 kDa (Transcript ID: ENST00000226413.5; Refseq: NM_000406.3). Fan et al. showed, due to the size of the gene, that the promoter and the 3′-UTR regions contain multiple transcription initiation sites and polyadenylation signals, respectively [24]. In 1997, Grose et al. showed the presence in the pituitary of a second smaller transcript generated by alternative splicing and encoding a protein of size 249 aa (Transcript ID: ENST00000000420975.2). Expression of this smaller protein was shown to be inhibitory to the mechanism of GnRHR signaling [28]. The 5′ UTR is found in exon 1 as well as the first 522 nucleotides of the coding sequence, which encode the first three transmembrane (TM) domains and a part of the fourth TM domain. Exon 2 encodes the following 220 nucleotides of the coding sequence, which includes the rest of the fourth TM and the fifth TM domain. Finally, exon 3 consists of the remaining 245 nucleotides of the coding sequence and the 3′ UTR [24,25,26] (Figure 1A).

### 2.2. Protein Structure of GnRHR

The GnRHR, as a member of the GPCR protein family, is composed of seven alpha-helical transmembrane (TM) domains that span the lipid bilayer of the cell membrane. These seven TMs are designated TM1 to TM7 and are connected by three intracellular (IL1–3) and three extracellular loops (EL1–3). The amino-terminal end (NH_2_) is on the extracellular side and the carboxyl-terminal end is on the intracellular side [14,29] (Figure 1B). TMs form a barrel-like structure that creates a hydrophobic core, allowing the receptor to integrate itself in the lipid bilayer of the cell membrane. This provides the structural framework of the receptor’s function, allowing it to interact extracellularly with GnRH ligands and intracellularly with G proteins [30,31]. The extracellular loops of the GnRHR help in the stability of the ligand-receptor complex. Specific amino acid residues of the extracellular loops of the receptor form a ligand-binding pocket that allows recognition and binding of the GnRH ligand with high specificity and its partial entry to the transmembrane part of the receptor [31]. Specifically, amino acid residues Asp at position 98, Trp at position 101, Asn at position 102, Lys at position 121, and Asp at position 302 were identified as important residues for ligand binding [31,32,33,34,35] (Figure 1B). The GNRHR intracellular loops are involved in G protein coupling. They contain specific sequence motifs that interact with G proteins, facilitating the activation of downstream signaling pathways. Moreover, intracellularly, the GnRHR differs from other GPCRs in a number of ways, including the absence of the carboxyl COOH terminal tail. This domain is regularly anchored to the membrane in other GPCRs [36] and plays an essential part in short-term desensitization caused by ligand-stimulated phosphorylation of Ser/Thr residues [37]. In GnRHR-mediated inositol phosphate production, the immediate desensitization is not observed, which is in line with the absence of a carboxy-terminal tail. Another difference of GnRHR compared to GPCRs is the substitution of Tyr with Ser, at position 140, in the highly conserved GPCR motif Asp-Arg-Tyr found at the junction of TM3 and the second intracellular loop [38,39,40]. This motif’s Asp and Arg residues have been related to the interaction of several GPCRs to their corresponding G proteins [41]. In addition, the substitution of Ser140 for Tyr had no effect on the GnRHR’s coupling to cytoplasmic G proteins [42,43]. The Tyr140 mutant receptor, on the other hand, increased the degree of receptor internalization and agonist-binding affinity, indicating that this substituted residue has mild impact on GNRHR structure [43]. The conserved residues ile135, ile143, and leu147 in the second intracellular loop of GnRHR play an important role in G protein coupling, validating the significance of this loop for the coupling with G proteins [43,44].

### 2.3. Signal Transduction Pathways of GNRHR

**Figure 2:**
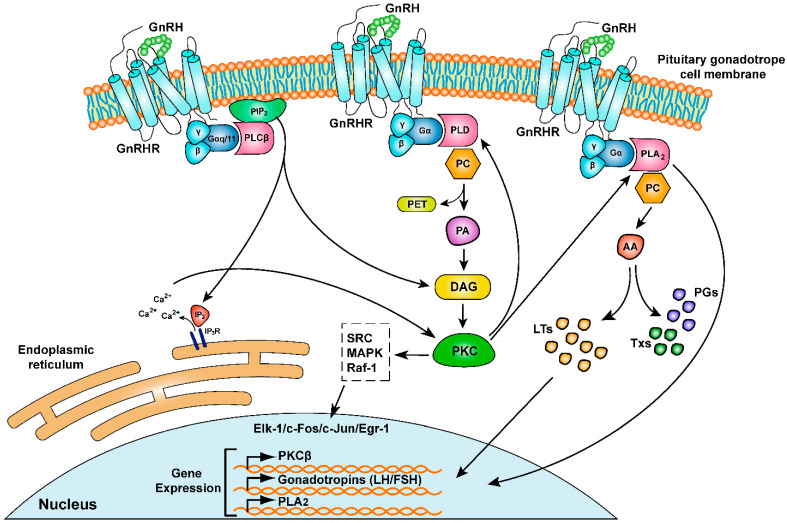
Signal transduction pathways of GnRHR. Schematic illustration of GnRHR activation by GnRH ligand binding to the pituitary gonadotrope cell leading to the expression of the gonadotropin genes, LH/FSH. When GnRH binds to its receptor (GnRHR), structural changes occur in both the receptor and the heterotrimeric GTP-binding proteins (G proteins), specifically the Gα_q/11_ subunit. This leads to a shift in the affinity of G protein for GTP over GDP, causing the Gα subunit to detach from the Gβγ dimer. The Gα_q/11_ subunit primarily triggers the phospholipase Cβ (PLCβ) pathway, leading to the hydrolysis of phosphatidylinositol 4,5 bisphosphate (PIP_2_) to inositol 1,4,5-trisphosphate (IP_3_) and diacylglycerol (DAG). IP_3_ induces Ca^2+^ release from the endoplasmic reticulum, whereas DAG, along with Ca^2+^, activates protein kinase C (PKC). In addition, GnRH binding to the GnRHR causes delayed activation of phospholipase D (PLD) that hydrolyzes the membrane phosphatidylcholine (PC), generating phosphatidylethanol (PET) and phosphatidic acid (PA) that cause DAG production, resulting in prolonged PKC activation. PKC then triggers the activation of various kinases, including Raf-1, src, and mitogen-activated protein kinases (MAPKs), which ultimately activate transcription factors such as Elk-1, Egr-1, c-Fos and c-Jun, leading to the expression of genes involved in gonadotropin production. The pathway also involves the activation of phospholipase A_2_ (PLA_2_) that hydrolyses PC to produce arachidonic acid (AA) which is converted to prostaglandins (PGs), thromboxanes (Txs), and leukotrienes (LTs) with the latter playing a role in gene activation and gonadotropin production.

GnRH and its receptor are differently distributed in the various tissues where they are detected. Consequently, the signaling pathways activated by the binding of GnRH to its receptor, in order to transmit extracellular information intracellularly, are strongly influenced by the type of cells in which they occur [45]. In pituitary gonadotrope cells, binding of GnRH to its receptor results in structural changes in both the receptor and the heterotrimeric GTP-binding proteins (G proteins). Heterotrimeric G proteins comprise three subunits: Gα, Gβ, and Gγ [46,47,48,49,50]. G proteins are differentiated into several subclasses based on differences in the structure of the Gα subunit. In mammals, Gα subunits belong to several subtypes: Gα_s_, Gα_q/11_, Gα_12/13_, Gα_i/o_, and Gα_t_ [51,52]. In pituitary gonadotrope cells, GnRHR is associated with the Gα_q/11_ subunit and, by few studies, with the Gα_s_ subunit [46]. The structural changes that occur in the heterotrimeric G protein result in an altered affinity for GDP and its replacement by GTP. This change promotes the detachment of the Gα subunit from the heterotrimer and its separation from the Gβγ dimer subunits, which remain as one [49,53]. Initial studies on the GnRH signaling pathway in pituitary gonadotrope cells revealed that the Gα_q/11_ subunit intracellularly triggers a series of signaling events by binding to phospholipase Cβ (PLCβ) [54] (Figure 2). Further studies showed that the Gβγ dimer could also bind to and activate PLCβ. It was also shown that upon prolonged GnRH stimulation, other factors such as the phospholipase A2 (PLA_2_) and phospholipase D (PLD) can be stimulated by either Gα or Gβγ [55]. The primary signaling pathway of GnRH’s response is through the phospholipase PLCβ which catalyzes the enzymatic hydrolysis of the membrane phospholipid phosphatidylinositol 4,5 bisphosphate (PIP_2_), synthesizing the inositol 1,4,5-trisphosphate (IP_3_) and diacylglycerol (DAG) [46,56]. IP_3_ activates the release of Ca^2+^ into the cytosol through its binding to IP_3_ receptors on the membrane of the endoplasmic reticulum, which act as Ca^2+^ channels [57,58]. Due to GnRH activation, Ca^2+^ accumulation together with DAG are the major causes of the production and secretion of gonadotropins in gonadotrope cells by activating protein kinase C (PKC) [59,60] (Figure 2). In gonadotrope cells, as mentioned above, GnRH binding to GnRHR also causes a delayed activation of PLD. PLD hydrolyzes the membrane phosphatidylcholine (PC) generating phosphatidylethanol (PET) and phosphatidic acid (PA) that eventually result in the production of DAG. The production of DAG by this pathway causes a sustained and prolonged activation of PKC, which has its two isoforms α and βII phosphorylate PLD as a positive feedback mechanism [61,62,63] (Figure 2). PKC activation also induces the activation of fibrosarcoma protein kinase 1 (Raf-1), protein tyrosine kinase src, and certain mitogen-activated protein kinases (MAPKs) [64,65,66]. These kinases act through the MAPK signaling pathway in which the end result is the phosphorylation and activation of transcription factors, including Elk-1, Egr-1, c-Fos, and c-Jun, which have a positive effect on the expression of gonadotropin kinase subunit α and PLA_2_ [56,64,67] (Figure 2). In addition, the PLA_2_ phospholipase produces arachidonic acid (AA), which is used as a substrate in a number of intracellular signals. AA is converted into leukotrienes by lipoxygenase, which in elevated concentrations are involved in the gene activation of PKCβ and the gonadotropins α subunit [68,69] (Figure 2). In summary, all signaling pathways activated by the binding of GnRH to the GnRHR receptor are interconnected and eventually result in the production and secretion of gonadotropins, LH, and FSH [5]. Moreover, due to the different pulse frequencies of GnRH secretion, different subunits of gonadotropins are produced. Specifically, when gonadotrope cells are exposed to an increased pulse frequency of GnRH, there is an induction in the production and release of the α subunit of gonadotropin and the β subunit of LH. Conversely, when cells are exposed to a low pulse frequency of GnRH, there is induction in the synthesis and release of the β subunit of FSH [70] (Figure 2).

### 2.4. Tissue Expression of GnRHR in Humans

Pituitary gland is the main tissue with the highest expression of GNRHR. Initial localization studies regarding the GnRHR transcript(s) identified three different transcripts. The primary transcript is correctly spliced and encodes the full-length protein [25,71]. The second transcript contains a 128-nucleotide deletion in exon 2 that causes alternative splicing, resulting in a truncated protein with a change in amino acid 174 and the addition of an extra 75 new amino acids. It is worth noting that this shorter transcript when expressed together with the full-length causes a dominant-negative effect preventing the wild-type protein from normally entering the cell membrane. The third transcript contains a 220-nucleotide deletion at exon 2 encoding a truncate protein with a size of 177 amino acids [28,72]. Immunoreactivity experiments demonstrated that GnRHR is specifically expressed in the gonadotrope, thyrotrope, and somatotrope cells of the pituitary gland [73,74]. GNRHR expression has been identified in other tissues other than the pituitary gland that are related or not to reproduction. Experiments in different ovarian cell types, as well as in several ovarian cancer lines, have shown in addition to GnRHR protein expression, the presence of GnRHR mRNA transcripts and the presence of binding sites for the GnRH ligand [75,76,77,78]. Binding sites for the GnRH ligand have also been found in uterus related cells, as well as in endometrial cancer lines. GnRHR transcripts have also been found in normal and neoplastic uterine cells [79,80]. GnRHR expression has also been observed in various placental cells such as cytotrophoblasts and syncytiotrophoblasts [81]. Moreover, GnRHR expression has also been detected in normal and neoplastic prostate gland cells [82,83] in various breast cancer cell lines [84,85,86] and in cells and cell lines not associated with reproduction, i.e., heart, skeletal muscles, liver, kidney, peripheral blood mononuclear cells, and melanoma cells [45,87,88].

### 2.5. GnRHR Characterized Mutations

**Figure 3:**
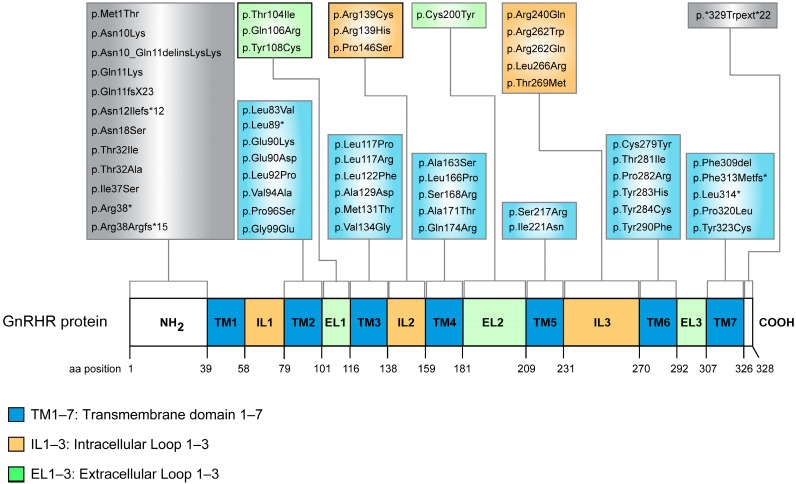
Disease-associated GnRHR mutations. Schematic illustration of the GnRHR protein with indication of the position of each mutation in the amino-terminal (NH_2_) tail, transmembrane (TM), intracellular (IL), extracellular (EL), and carboxy-terminal (COOH) domains.

Since 1997 when de Roux et al. identified the first mutation in the *GNRHR* gene in a family with hypogonadotropic hypogonadism (HH), several mutations in the gene have been described [89]. The mode of inheritance regarding mutations in the *GNRHR* follows the autosomal recessive manner and, to date, have been found in both the homozygous and compound heterozygous state. Furthermore, patients with HH have been found to carry, in a digenic mode of inheritance fashion, a heterozygous in the *GNRHR* and a second mutation in the second allele in one of the *ANOS1* (*KAL1*), *FGFR1*, *GNRH1*, *FGF8*, *PROK2*, *PROKR2*, *KISS1R*, *CHD7*, *TAC3*, and *TACR3* genes. All these genes have recently reported to be associated with HH [90,91,92,93]. It is worth mentioning that the above-mentioned pathogenic variants have also been associated with HH with phenotypes such as partial or total delayed puberty, infertility, and Kallmann syndrome. In HH cases, mutations in *GNRHR* account for 3.5–16% of sporadic cases and up to 40% of familial cases [91,94]. To date, 58 mutations have been reported including 48 missense, 3 nonsense, 5 frameshift, 1 in-frame, and 1 splice acceptor [89,90,95,96,97,98,99,100,101,102,103,104,105,106,107,108,109,110,111,112,113,114,115,116,117,118,119,120,121,122,123,124,125,126,127,128,129] (Table 1). Interestingly, all of the these mutations are localized in all regions of the receptor except the first transmembrane region (TM1), the first intracellular loop (IL1), and the third extracellular loop (EL3) (Figure 3). The mutations have been described as inactivating and cause an alteration in the function of the receptor either by reducing its expression, its localization, impairing ligand binding, and/or affecting signaling. It is worth mentioning that three of the identified mutations, p.Gln106Arg, p.Arg139His, and p.Arg262Gln, show an increased frequency number compared to the others. According to gnomAD browser (https://gnomad.broadinstitute.org/ (accessed on 2 October 2023)), the allele frequency for p.Gln106Arg is 0.002749, for p.Arg139His is 0.0001630, and for p.Arg262Gln is 0.001789, while allele frequencies for the remaining mutations range from 0.0001291 to 0.000004005. Due to this increased frequency, these three mutations have also been found in non-consanguineous families while the remaining mutations, when found in homozygous tissue, typically come from consanguineous families [122,130,131]. For this reason, in 2015, Choi et al. investigated the possibility of these mutations being inherited from a common ancestor. Indeed, it was proven so that all of the tested patients with these specific mutations shared a common haplotype, thus suggesting that they have been inherited from a common ancestor and behave as founder mutations [131]. Identification and characterization of the mutations will demonstrate the regions of GNRHR that are important for ligand binding, signaling, proper protein folding, and correct localization.

### 2.6. GnRHR Ligands: Agonists, Antagonists, and Pharmacoperones

The GNRHR is a key regulator of reproductive processes, and a wide range of ligands, including agonists, antagonists, and pharmacoperones, can control its function [23,132,133,134]. These compounds have various effects on the receptor, controlling the secretion of LH and FSH, all of which are important in the male and female reproduction process.

### 2.7. GnRHR Agonists

#### 2.7.1. Gonadotropin-Releasing Hormone (GnRH)

The endogenous GnRH, when bound to the GNRHR, functions as an agonist. GnRH is synthesized in the hypothalamus and serves as the catalyst for the pituitary gland’s pulsatile secretion of LH and FSH [45]. Its pulsatile secretion behavior is critical for reproductive process regulation.

#### 2.7.2. GnRH Analogues (GnRHa)

Synthetic GnRH analogues are used to treat infertility, endometriosis, uterine fibroids, precocious puberty, hypogonadotropic hypogonadism, and hormone-sensitive cancers of the breast in women and prostate in men [135,136,137,138,139,140,141,142]. In HH treatment, GnRHR analogues bind to GnRHR on the pituitary gland, leading to the initial release of LH and FSH. Upon administration of a GnRHR analogue, there is an initial “flare” effect, in which there is a brief surge of LH and FSH production. This flare can temporarily worsen symptoms in some individuals with HH. After the initial flare, continuous exposure to GnRHR analogue leads to desensitization of the pituitary gland. The pituitary becomes less responsive to GnRH, which results in a decrease in LH and FSH production. By initially stimulating the release of LH and FSH and subsequently downregulating their production, GnRHR analogues can help normalize sex hormone (estrogen and testosterone) levels over time. This process is critical for reproductive regulation due to the decrease in estrogen and testosterone levels in HH patients [135]. Examples of GnRHa used in therapy are the leuprorelin, goserelin, nafarelin, triptorelin, buserelin, and histrelin. In addition, deslorelin is used in veterinary medicine for a variety of purposes such as promoting ovulation and the treatment of high-risk pregnancies in animals. Gonadorelin is used in both humans and animals. In contrast to other GnRH analogues, which are used to inhibit LH and FSH secretion, deslorelin is associated with stimulation of LH secretion (Table 2).

### 2.8. GnRHR Antagonists

GnRH antagonists, contrary to GnRHa, directly block the GnRH receptor, and thus the action of GnRH, without the initial activation seen with agonists. The resulting LH suppression causes rapid reduction of the production of testosterone in the testes in men, and a reduction of estradiol and progesterone production from the ovaries in women. GnRH antagonists are capable of preventing gonadal sex hormone production and suppressing sex hormone levels [143]. In controlled ovarian stimulation protocols for in vitro fertilisation (IVF), this rapid blockade prevents premature LH surges [144]. GnRH antagonists are used in assisted reproductive technologies for fertility treatment as well as the treatment of conditions such as precocious puberty, endometriosis, uterine fibroids, and prostate cancer [137,138,139,140,145]. Many GnRH antagonists, like cetrorelix, degarelix, abarelix, and ganirelix, have a structure analogous to natural GnRH but have an antagonistic activity, whereas others, like elagolix, linzagolix, and relugolix, are non-peptide compounds (Table 3).

### 2.9. GnRHR Pharmacoperones

Pharmacological chaperones or Pharmacoperones are small molecules that bind to a target protein and correct or improve its folding, trafficking, or stability. These molecules have the ability to restore proper protein function, making them a potential therapy for genetic disorders caused by misfolded or impaired proteins [134]. In the case of GnRHR, specific genetic mutations can cause its misfolding and retention in the endoplasmic reticulum, preventing it from reaching the cell membrane. Pharmacoperones can therefore successfully rescue mutant GNRHRs that would otherwise be non-functional, helping to properly fold the receptor and thus, traffic it to the cell membrane, while restoring its ability to respond to GnRH [146,147]. All described GnRHR pharmacoperones act as receptor antagonists. The first study was conducted using four different pharmacoperones; IN3, Q89, A177775, and TAK-013, and tested their ability to restore GnRHR function in the COS-7 cell line that expressed the mutant GnRHR. All four pharmacoperones were successful in restoring cell-surface expression and stimulating constitutive activity [148]. The properties of pharmacoperone IN3 were further studied in transgenic mice with a hypogonadotropic hypogonadism phenotype due to the GnRHR p.Glu90Lys mutation. After a 30-day treatment with IN3, male mice showed elevated sperm concentration, positive changes in sperm morphology, and increased expression of steroidogenic enzymes [148]. This approach holds promise for the treatment of genetic disorders caused by mutations in GNRHR, such as congenital hypogonadotropic hypogonadism (CHH).

### 2.10. Clinical Implications of Mutated GnRHR

The main feature in patients carrying mutations in GnRHR is normosmic hypogonadotropic hypogonadism. These patients exhibit a variety of clinical symptoms characterized by different phenotypic diagnosis and/or different evaluation at age of diagnosis. Spontaneous pulsality of gonadotropins is not normal in patients with GnRHR mutations, showing reduced intensity but normal frequency and absence of pulsality of LH and FSH secretion [89,104,110,119,149,150].

In HH, the HPG axis is disrupted due to dysfunction at the hypothalamic and/or pituitary level. This disorder leads to a deficiency of the key hormones GnRH, LH, and FSH, resulting in a cascade of effects. The hypothalamus fails to produce and release GnRH or does so inadequately. This means that there is insufficient signal to the pituitary gland to stimulate the release of LH and FSH. Regardless of the specific point of dysfunction, the end result is low levels of LH and FSH in the bloodstream. With low levels of LH and FSH, the gonads receive inadequate stimulation. In males, this result in decreased testosterone production, while in females, there is a lack of proper ovarian stimulation for the production of estradiol and progesterone [151,152,153,154]. As a result, individuals with HH have low levels of sex hormones, leading to various clinical features such as delayed or absent puberty, infertility, and absence of secondary sexual characteristics.

More specifically, in childhood and adolescence, HH is characterized by delayed or completely absent of puberty. Girls with HH often do not show the expected signs of puberty at the typical age, such as breast development and minimal body hair growth, while boys show absence of facial and body hair growth, impaired testicular development, and limited muscle growth. In addition, delayed growth and development in children and adolescents, defined by delayed bone age compared to chronological age, can result in shorter stature and slower growth rates compared to their peers. As a consequence of insufficient sex hormone production, individuals with HH in this age group are typically infertile. This is due to the gonads’ inability to produce mature eggs or sperm necessary for reproduction. Adolescents with HH may experience several symptoms related to low levels of sex hormones. In boys, this may include low libido, erectile dysfunction, and fatigue. Girls may experience menstrual irregularities, including primary or secondary amenorrhea (absence of menstruation), as well as symptoms such as mood changes and fatigue. The uterus is the size of that at the prepubertal stage and the ovaries are small or absent due to lack of follicular stimulation. Furthermore, the delayed or absent puberty and the absence of typical secondary sexual characteristics can have a significant psychosocial impact on adolescents. This may lead to feelings of self-consciousness, reduced self-esteem, and emotional challenges [104,114,117,119,125]. In adulthood, HH presents with a range of clinical characteristics that reflect the deficiency of sex hormones, particularly testosterone in males and estradiol in females. These clinical characteristics can vary depending on the underlying cause and the individual’s specific case. In males, HH can result in the loss of secondary sexual characteristics, such as decreased facial and body hair growth, reduced muscle mass, gynecomastia, microphallus, and a decrease in the size of the testes [89,110,113,114,119,149]. In females, there may be a loss of breast development, changes in body fat distribution, and the absence of typical female secondary sexual characteristics. Infertility is a significant concern for individuals with HH in adulthood. The lack of adequate sex hormone production can lead to the inability to conceive naturally. Both men and women with HH may experience infertility, and assisted reproductive technologies may be necessary to achieve pregnancy [96,104,110,111,150,155]. However, hormonal treatment with human chorionic gonadotropin resulted in production of normal sperm counts leading to successful conception and pregnancy [89,149,150,156]. Sexual dysfunction is a common feature of HH in both men and women. In males, this may manifest as erectile dysfunction and reduced libido. In females, it can lead to decreased sexual desire and vaginal dryness. Low sex hormone levels in HH can lead to symptoms of fatigue, reduced energy levels, and a general sense of sickness. This can affect an individual’s quality of life and overall well-being. Men with HH may experience symptoms such as hot flashes (similar to those seen in menopause), changes in body composition, and a decrease in strength and stamina. The lack of testosterone can result in muscle weakness and an increased risk of osteoporosis. Moreover, low testosterone levels in men with HH can be associated with metabolic effects, including increased body fat, insulin resistance, and potentially an increased risk of cardiovascular disease. Women with HH may have irregular menstrual cycles, amenorrhea (absence of menstruation), and symptoms like hot flashes, night sweats, and changes in bone density [157,158,159]. Although most patients with mutations in GnRHR are thought to have hypogonadism early at birth, this is diagnosed later when clinical symptoms are more prominent [160]. The underlying causes of CHH can vary and may include genetic mutations, congenital abnormalities, or disruptions in the hypothalamic-pituitary-gonadal axis. It is important to note that early diagnosis and management of HH in childhood and adolescence are crucial to address these clinical characteristics effectively. Treatment typically involves hormone replacement therapy to induce and support the development of secondary sexual characteristics and normal growth [159,161,162]. Similarly, early diagnosis and management of HH in adulthood are important to address the clinical characteristics effectively. Treatment often involves hormone replacement therapy (e.g., testosterone replacement in men or estradiol/progesterone replacement in women) to correct hormonal imbalances and alleviate associated symptoms. Endocrinologists and specialists in hormonal disorders play a key role in the diagnosis and management of HH in children, adolescents, and adults.

## 3. Conclusions

In summary, GnRH and its receptor orchestrate complex signaling pathways, influenced by tissue-specific distribution. These pathways involve heterotrimeric G proteins, ultimately leading to gonadotropin production. Various ligands, including agonists, antagonists, and pharmacoperones, modulate the receptor’s function. Genomic and protein structures reveal critical regions and mutations associated with reproductive disorders. GnRH receptor expression extends to diverse tissues. Clinical implications encompass hypogonadotropic hypogonadism with symptoms affecting both genders. Future perspectives include tailored therapies for GnRHR mutations and advancing precision medicine in reproductive disorders. Current research may reveal additional roles for GNRHR in non-reproductive tissues, expanding our understanding of their broader physiological impact and potential therapeutic applications beyond reproduction.

## Figures and Tables

**Table 1:** Disease-associated mutations found in GnRHR.

cDNA (NM_000406.3)	Protein (NP_000397.1)	Molecular Consequence	Region Affected	Phenotype	Classification	Reference
c.2T>C	p.Met1Thr	Missense	NH_2_ tail	Hypogonadotropic hypogonadism	Pathogenic	[103]
c.30T>A	p.Asn10Lys	Missense	NH_2_ tail	Hypogonadotropic hypogonadism	Likely Pathogenic	[117]
c.30_31delinsAA	p.Asn10_Gln11delinsLysLys	Missense	NH_2_ tail	Hypogonadotropic hypogonadism	Pathogenic	[117]
c.31C>A	p.Gln11Lys	Missense	NH_2_ tail	Hypogonadotropic hypogonadism.	Likely Pathogenic	[97]
c.32delA	p.Gln11fsX23	Frameshift	NH_2_ tail	Hypogonadotropic hypogonadism	Pathogenic	[103]
c.35delA	p.Asn12Ilefs*12	Frameshift	NH_2_ tail	Hypogonadotropic hypogonadism	Pathogenic	[128]
c.53A>G	p.Asn18Ser	Missense	NH_2_ tail	Hypogonadotropic hypogonadism	Likely Pathogenic	[115]
c.94A>G	p.Thr32Ala	Missense	NH_2_ tail	Hypogonadotropic hypogonadism	Pathogenic	[106]
c.95 C>T	p.Thr32Ile	Missense	NH_2_ tail	Hypogonadotropic hypogonadism	Pathogenic/Likely Pathogenic	[103]
c.110T>G	p.Ile37Ser	Missense	NH_2_ tail	Hypogonadotropic hypogonadism	Pathogenic	[115]
c.112C>T	p.Arg38*	nonsense	NH_2_ tail	Hypogonadotropic hypogonadism	Pathogenic	[127]
c.113_114insG	p.Arg38Argfs*15	Frameshift	NH_2_ tail	hypogonadotropic hypogonadism	Pathogenic	[115]
c.247C>T	p.Leu83Val	Missense	TM2	Hypogonadotropic hypogonadism	Pathogenic	[126]
c.266T>A	p.Leu89*	nonsense	TM2	Hypogonadotropic hypogonadism	Pathogenic	[107]
c.268G>A	p.Glu90Lys	Missense	TM2	Hypogonadotropic hypogonadism	Pathogenic/Likely Pathogenic	[125]
c.270G>C	p.Glu90Asp	Missense	TM2	Hypogonadotropic hypogonadism	Likely Pathogenic	[115]
c.275T>C	p.Leu92Pro	Missense	TM2	Hypogonadotropic hypogonadism	Likely Pathogenic	[124]
c.281T>C	p.Val94Ala	Missense	TM2	Hypogonadotropic hypogonadism	Likely Pathogenic	[123]
c.286C>T	p.Pro96Ser	Missense	TM2	Hypogonadotropic hypogonadism	Likely Pathogenic	[103]
c.296G>A	p.Gly99Glu	Missense	TM2	Hypogonadotropic hypogonadism	Likely Pathogenic	[122]
c.311C>T	p.Thr104Ile	Missense	EL1	Hypogonadotropic hypogonadism	Likely Pathogenic	[121]
c.317A>G	p.Gln106Arg	Missense	EL1	Hypogonadotropic hypogonadism	Pathogenic/Likely Pathogenic	[89]
c.323 A>G	p.Tyr108Cys	Missense	EL1	Hypogonadotropic hypogonadism	Likely Pathogenic	[121]
c.350T>C	p.Leu117Pro	Missense	TM3	Delayed Puberty	Likely Pathogenic	[103]
c.350T>G	p.Leu117Arg	Missense	TM3	Hypogonadotropic hypogonadism	Pathogenic/Likely Pathogenic	[95]
c.364C>T	p.Leu122Phe	Missense	TM3	Hypogonadotropic hypogonadism	Pathogenic	[120]
c.386C>A	p.Ala129Asp	Missense	TM3	Hypogonadotropic hypogonadism	Pathogenic	[119]
c.392T>C	p.Met131Thr	Missense	TM3	Hypogonadotropic hypogonadism	Pathogenic	[95]
c.401T>G	p.Val134Gly	Missense	TM3	Hypogonadotropic hypogonadism	Pathogenic	[102]
c.415C>T	p.Arg139Cys	Missense	IL2	Hypogonadotropic hypogonadism	Pathogenic	[118]
c.416G>A	p.Arg139His	Missense	IL2	Hypogonadotropic hypogonadism	Pathogenic	[117]
c.436C>T	p.Pro146Ser	Missense	IL2	Hypogonadotropic hypogonadism	Likely Pathogenic	[116]
c.487G>T	p.Ala163Ser	Missense	TM4	Kallman syndrome	Likely Pathogenic	[115]
c.497T>C	p.Leu166Pro	Missense	TM4	Hypogonadotropic hypogonadism	Likely Pathogenic	[103]
c.504T>A	p.Ser168Arg	Missense	TM4	Hypogonadotropic hypogonadism	Pathogenic	[114]
c.511G>A	p.Ala171Thr	Missense	TM4	Hypogonadotropic hypogonadism	Pathogenic	[113]
c.521A>G	p.Gln174Arg	Missense	TM4	DSD	Likely Pathogenic	[112]
c.523-1G>A	-	Splice acceptor	-	Hypogonadotropic hypogonadism	Pathogenic	[111]
c.599G>A	p.Cys200Tyr	Missense	EL2	Hypogonadotropic hypogonadism	Pathogenic	[104]
c.651C>A	p.Ser217Arg	Missense	TM5	Hypogonadotropic hypogonadism	Pathogenic	[110]
c.662T>A	p.Ile221Asn	Missense	TM5	Hypogonadotropic hypogonadism	Hypogonadotropic hypogonadism	[109]
c.719G>A	p.Arg240Gln	Missense	IL3	Kallmann syndrome	Likely Pathogenic	[108]
c.784C>T	p.Arg262Trp	Missense	IL3	Hypogonadotropic hypogonadism	Likely Pathogenic	[107]
c.785G>A	p.Arg262Gln	Missense	IL3	Hypogonadotropic hypogonadism	Pathogenic/Likely Pathogenic	[89]
c.797T>G	p.Leu266Arg	Missense	IL3	Hypogonadotropic hypogonadism	Pathogenic/Likely Pathogenic	[104]
c.806C>T	p.Thr269Met	Missense	IL3	Hypogonadotropic hypogonadism	Pathogenic	[105,106]
c.836G>A	p.Cys279Tyr	Missense	TM6	Hypogonadotropic hypogonadism	Pathogenic	[104]
c.842C>T	p.Thr281Ile	Missense	TM6	Hypogonadotropic hypogonadism	Pathogenic	[103]
c.845C>G	p.Pro282Arg	Missense	TM6	Hypogonadotropic hypogonadism	Likely Pathogenic	[98]
c.847T>C	p.Tyr283His	Missense	TM6	Hypogonadotropic hypogonadism	Pathogenic	[102]
c.851A>G	p.Tyr284Cys	Missense	TM6	Hypogonadotropic hypogonadism	Likely Pathogenic	[101]
c.869A>T	p.Tyr290Phe	Missense	TM6	Hypogonadotropic hypogonadism	Likely Pathogenic	[100]
c.924_926delCTT	p.Phe309del	In frame deletion	TM7	Delayed Puberty	Likely Pathogenic	[99]
c.937_947del	p.Phe313Metfs*3	Frameshift	TM7	hypogonadotropic hypogonadism	Pathogenic	[90]
c.941T>A	p.Leu314*	nonsense	TM7	Hypogonadotropic hypogonadism	Pathogenic	[96]
c.959C>T	p.Pro320Leu	Missense	TM7	Hypogonadotropic hypogonadism	Pathogenic	[97]
c.968A>G	p.Tyr323Cys	Missense	TM7	Hypogonadotropic Hypogonadism	Pathogenic	[98]
c.987A>G	p.X329WextX22	Frameshift	COOH tail	Hypogonadotropic hypogonadism	Likely Pathogenic	[95]
Dup of Exon 1	-	Exon Duplication	-	Hypogonadotropic hypogonadism	Pathogenic	[129]
Del of Exon 2	-	Exon Deletion	-	Hypogonadotropic hypogonadism	Pathogenic	[129]

**Table 2:** GnRHR agonists with clinical or veterinary applications.

Name	Brand Name	PubChem CID	Medical Applications
GnRH	-		Natural ligand of GnRHR
Buserelin	Suprefact	50225	Breast cancer, Endometrial hyperplasia, Endometriosis, Female infertility, Prostate cancer, Uterine fibroids
Goserelin	Zoladex	5311128	Breast cancer, Endometriosis, Female infertility, Prostate cancer, Uterine fibroids, Uterine hemorrhage
Histrelin	Vantas, Supprelin	25077993	Precocious puberty, Prostate cancer
Leuprorelin	Lupron	657181	Breast cancer, Endometriosis, Menorrhagia, Precocious puberty, Prostate cancer, Uterine fibroids
Nafarelin	Synarel	25077405	Precocious puberty, Endometriosis
Triptorelin	Decapeptyl	25074470	Breast cancer, Endometriosis, Female infertility, Precocious puberty, Prostate cancer, Uterine fibroids
Gonadorelin	Factrel	638793	Cryptorchidism, Delayed puberty, Hypogonadotropic hypogonadism, Veterinary medicine (assisted reproduction)
Lecirelin	Dalmarelin	66577115	Veterinary medicine (assisted reproduction)
Peforelin	Maprelin	16197823	Veterinary medicine (assisted reproduction)
Azagly-nafarelin	Gonazon	156613532	Veterinary medicine (assisted reproduction)
Deslorelin	Ovuplant, Suprelorin	25077495	Veterinary medicine (assisted reproduction)
Fertirelin	Ovalyse	188304	Veterinary medicine (assisted reproduction)

**Table 3:** GnRHR antagonists with clinical applications.

Name	Brand Name	Molecule Status	PubChem CID	Medical Applications
Abarelix	Plenaxis	Peptide	16131215	Prostate cancer
Cetrorelix	Cetrotide	Peptide	25074887	Female infertility
Degarelix	Firmagon	Peptide	6136245	Prostate cancer
Ganirelix	Orgalutran	Peptide	16130957	Female infertility
Linzagolix	KLH-2109, OBE-2109	Non-peptide	16656889	Endometriosis, Uterine fibroids
Relugolix	Relumina	Non-peptide	10348973	Uterine fibroids, Prostate cancer
Elagolix	Orilissa	Non-peptide	11250647	Endometriosis, Uterine fibroids

## Data Availability

No new data were created or analyzed in this study. Data sharing is not applicable to this article.
